# DNA and histone deacetylases as targets for neuroblastoma treatment

**DOI:** 10.2478/v10102-010-0010-6

**Published:** 2010-06

**Authors:** Marie Stiborová, Jitka Poljaková, Tomáš Eckschlager, Rene Kizek, Eva Frei

**Affiliations:** 1 Department of Biochemistry, Faculty of Science, Charles University, Prague, Czech Republic; 2 Department of Pediatric Hematology and Oncology, 2nd Medical School, Charles University and University Hospital Motol, Prague, Czech Republic; 3 Department of Chemistry and Biochemistry, Faculty of Agronomy, Mendel University of Agriculture and Forestry, Brno, Czech Republic; 4 Division of Preventive Oncology, National Center for Tumor Diseases, German Cancer Research Center, Heidelberg, Germany

**Keywords:** neuroblastoma, DNA-damaging anticancer drugs, inhibitors of histone deacetylases, mechanisms of acticancer effects of drugs

## Abstract

Neuroblastoma, a tumor of the peripheral sympathetic nervous system, is the most frequent solid extra cranial tumor in children and is a major cause of death from neoplasia in infancy. Still little improvement in therapeutic options has been made, requiring a need for the development of new therapies. In our laboratory, we address still unsettled questions, which of mechanisms of action of DNA-damaging drugs both currently use for treatment of human neuroblastomas (doxorubicin, cis-platin, cyclophosphamide and etoposide) and another anticancer agent decreasing growth of neuroblastomas *in vitro*, ellipticine, are predominant mechanism(s) responsible for their antitumor action in neuroblastoma cell lines *in vitro*. Because hypoxia frequently occurs in tumors and strongly correlates with advanced disease and poor outcome caused by chemoresistance, the effects of hypoxia on efficiencies and mechanisms of actions of these drugs in neuroblastomas are also investigated. Since the epigenetic structure of DNA and its lesions play a role in the origin of human neuroblastomas, pharmaceutical manipulation of the epigenome may offer other treatment options also for neuroblastomas. Therefore, the effects of histone deacetylase inhibitors on growth of neuroblastoma and combination of these compounds with doxorubicin, cis-platin, etoposide and ellipticine as well as mechanisms of such effects in human neuroblastona cell lines *in vitro* are also investigated. Such a study will increase our knowledge to explain the proper function of these drugs on the molecular level, which should be utilized for the development of new therapies for neuroblastomas.

## Introduction

Neuroblastoma, a tumor of the peripheral sympathetic nervous system, is the most frequent solid extra cranial tumor in children and is a major cause of death from neoplasia in infancy (Maris and Mathay, 1999; Schwab, 1999). These tumors are biologically heterogeneous, with cell populations differing in their genetic programs, maturation stage and malignant potential (Brodeur, 2003). Neuroblastoma consists of two principal neoplastic cells (Voigt *et al*., 2000; Hopkins-Donaldson *et al*., 2002): i) neuroblastic or N-type: undifferentiated, round and small cells with scant cytoplasm; and ii) stromal or S-type: large hyaline, flattened and adherent differentiated cells. As neuroblastoma cells seem to have the capacity to differentiate spontaneously *in vivo* and *in vitro* (Morgenstern *et al*., 2004), their heterogeneity could affect treatment outcome, in particular the response to apoptosis induced by chemotherapy. Neuroblastoma may regress spontaneously in infants, mature to benign ganglioneuromas in older children, or grow relentlessly and be rapidly fatal (Brodeur, 2003). Approximately 40% of all patients with neuroblastoma belong to the high-risk group (high-risk neuroblastoma), and therapeutic improvements in the past decade have not substantially improved their outlook in compared to other pediatric malignancies (Westermann and Schwab, 2002). Prognosis of high-risk neuroblastomas is poor, because drug resistance arises in the majority of those patients, initially responding to chemotherapy, in spite of intensive therapy including megatherapy with subsequent hematopoietic progenitor cell transplantation, biotherapy and immunotherapy (Brodeur, 2003). Treatment of older children with widely disseminated neuroblastoma remains one of the greatest challenges for pediatric oncologists. However, still little improvement in therapeutic options has been made in the last decade, requiring a need for the development of new therapies.

## Drug-resistance in neuroblastomas

Drug-resistance in neuroblastomas might be caused by multiple factors, including overexpression of the genes for the multidrug-resistance-associated protein (MRP) and *mdr-1* gene-encoded P-170 glycoprotein (Pgp) (Bates *et al*., 1991; Bader *et al*., 1999). High levels of MRP gene expression are frequently found in neuroblastoma resistant to chemotherapeutic agents and are proven to correlate with poor outcome in patients with neuroblastoma (Norris *et al*., 1996; Peaston *et al*., 2001), whereas the contribution of *mdr1* gene expression to the biology of childhood neuroblastomas is still a matter of debate (Kurowski and Berthold, 1998, Kucerova *et al*., 2001; de Cremoux *et al*., 2007). Drug-resistance could be also caused by changes in apoptotic pathways. For example, mutations in p53 or overexpression of Bcl-2, two regulators of apoptosis, contribute to neoplastic transformation by blocking apoptosis (Lara-Bohórquez *et al*., 2008). Protection of cell death by Bcl-2 does not require participation of another apoptotic regulator Bax, but it is most effective when Bcl-2 is in excess of Bax (Otter *et al*., 1998; Uccini *et al*., 2006). Chemoresistance is usually not caused by a single mechanism, but it is complex phenomenon (Bedrnicek *et al*., 2005). Recent studies have provided a link between the malignant and drug-resistant phenotypes, indicating that in addition to the development of drug resistance, chemotherapy of tumors may cause various changes in their biological characteristics, including increased malignancy and metastatic potential (Emanuel *et al*., 1999; Kotchetkov *et al*., 2005).

## Ellipticine as a promising drug for neuroblastoma treatment

**Figure 1:**
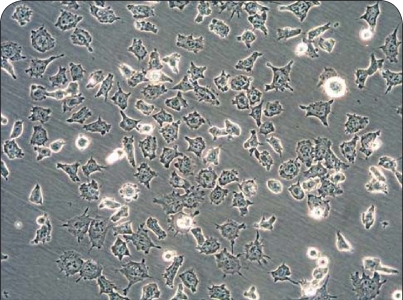
Neuroblastoma cell lines UKF-NB-4 (200-fold magnification) [adapted from reference (Poljakova *et al*., 2009)].

**Figure 2:**
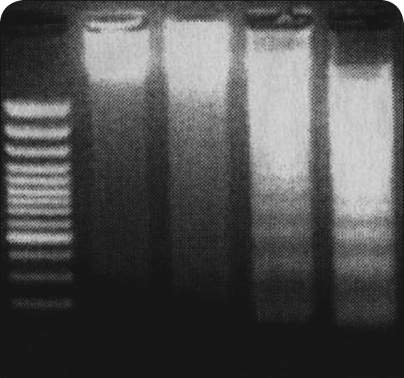
The induction of apoptosis in ellipticine-treated neuroblastoma UKF-NB-4 cells. The DNA fragmentation was assessed by gel electrophoresis. Cells were treated with vehicle and ellipticine for 48 h, and then the fragmentation of DNA was assessed by agarose gel electrophoresis. (Lane 1) marker; (lane 2) UKF-NB-4 cells treated with vehicle alone (DMSO); (lane 3) 0.1 µM ellipticine, (lane 4) 1 µM ellipticine, and (lane 5) 10 µM ellipticine [adapted from reference (Poljakova *et al*., 2009)].

**Figure 3:**
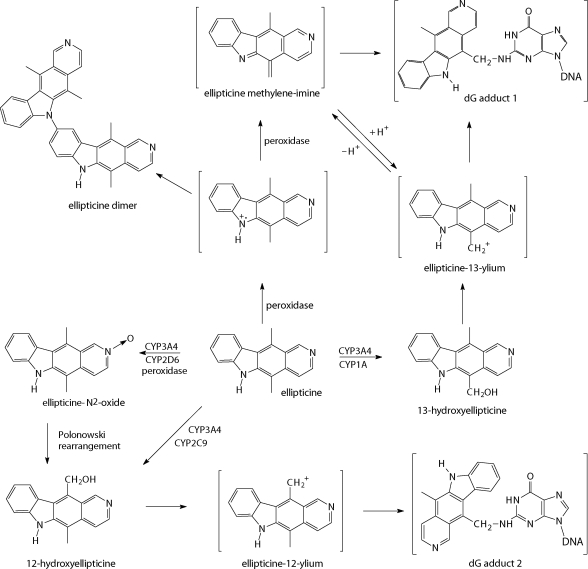
Scheme of the metabolism of ellipticine by peroxidases and human CYPs showing the characterized metabolites and those proposed to form DNA adducts. The compounds shown in brackets are the hypothetical electrophilic metabolites postulated as ultimate arylating species or the postulated *N* ^2^-deoxyguanosine adducts.

Even though currently used cytostatics, including cis-platin, etoposide, doxorubicin, vincristine and cyclophosphamide (Maris *et al*., 2007) are effective for treatment of neuroblastomas, they failed in their actions for a variety of patients. Hence, in the past two decades, attempts have been made to improve outcomes in high-risk patients by delivering intensive induction therapy. Recently, we have suggested novel treatment of neuroblastomas, utilizing a drug targeting DNA, plant alkaloid ellipticine. We have found that treatment of human neuroblastoma IMR-32, UKF-NB-3 and UKF-NB-4 cell lines (Figure 1) with this agent resulted in strong inhibition of cell growth, followed by induction of apoptosis (Figure 2) (Poljaková *et al*., 2008; 2009). These effects were associated with formation of two covalent ellipticine-derived DNA adducts, identical to those formed by the cytochrome P450- and peroxidase-mediated ellipticine metabolites, 13-hydroxy- and 12-hydroxyellipticine (Figure 3) (Stiborová *et al*., 2001; 2004; 2007a). In addition, besides the formation of such covalent ellipticine-derived DNA adducts, participation of the mechanisms in ellipticine toxicity to neuroblastoma, such as intercalation into DNA (Auclair, 1987; Singh *et al*., 1994), and inhibition of DNA topoisomerase II activity (Auclair, 1987; Monnot *et al*., 1991; Fossé *et al*., 1992; Froelich-Ammon *et al*., 1995] that were found to be additional DNA-mediated mechanisms of ellipticine antitumor, mutagenic and cytotoxic activities [for a summary see (Stiborová *et al*., 2001; 2006; 2010)], cannot be excluded. Therefore, ellipticine and/or its more effective derivatives seem to be promising drugs for future clinical applications (Poljaková *et al*., 2009). This suggestion, however, needs to be confirmed by further investigations including the *in vivo* studies.

Hypoxia frequently occurs in tumors because of their fast growth and inadequate vascularisation. It strongly correlates with advanced disease and poor outcome caused by chemoresistance. The hypoxia inducible factor (HIF) is a transcription factor, which seems to be important for cells to adapt to hypoxia. Hypoxia-induced chemoresistance to cis-platin and doxorubicin in human non-small cell lung cancer cells is through the HIF pathway and may be reversed by postransriptional blocking of this factor. (Song *et al*., 2006). However, even though this and other factors were suggested to influence cytostatic-induced apoptosis of cancer cells and to contribute to induce chemoresistance of cells to drugs, their actual roles in neuroblastomas remain still to be investigated. In the case of the ellipticine toxic activity to neuroblastoma cells, hypoxic cell culture conditions resulted in a decrease in ellipticine toxicity to these cells. One of the reasons causing this effect might follow from a decrease in formation of ellipticine-derived DNA adducts in neuroblastoma cells, but exact mechanisms of such effects still await further investigations (Poljaková *et al*., 2009).

Most of the drugs shown to be toxic to neuroblastomas and may be used for their treatment (doxorubicin, cisplatin, etoposide, cyclophosphamide and ellipticine) are known to be the DNA-damaging agents. Their effects include non-covalent DNA intercalation, inhibition of topoisomerase II, formation of covalent DNA adducts, DNA-DNA cross-links and/or free radical effects on DNA [for a summary see (Klener, 1996)]. The question, however, arises on the real contribution of these effects to overall cytotoxicity of these drugs to neuroblastoma cells. At the present time it is not known, whether these individual effects synergize to contribute to antitumor and cytostatic activities of these drugs to neuroblastoma cells. Moreover, it is also not possible to demonstrate which of these effects predominate in their cytotoxic activities.

## Combination of DNA-damaging drugs and epigenetics-based therapy with histone deacetylase inhibitors may be a promising therapy for neuroblastomas

**Figure 4:**
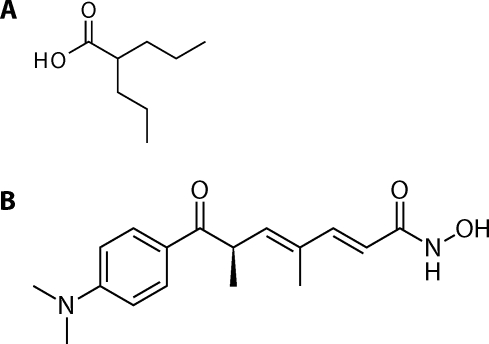
Valproic acid (**A**) and trichostatin A (**B**).

The use of potent epigenetics-based therapy for cancer cells might restore the abnormally regulated epigenomes to a more normal state through epigenetic reprogramming (Witt *et al*., 2009). Thus, epigenetic therapy may be a promising and potent treatment for human neoplasia. Because the epigenetic structure of DNA and its lesions play a role in the origin of human neuroblastomas, pharmaceutical manipulation of the epigenome may offer other treatment options also for neuroblastomas (Furchert *et al*., 2007). Indeed, former studies demonstrated the cytotoxicity of a panel of histone deacetylases inhibitors to neuroblastomas, resulting in growth inhibition of these tumor cells (Cinatl *et al*., 1996; Michaelis *et al*., 2004, 2007; Furchert *et al*., 2007). Histone deacetylases and histone acetyl transferases modify histone proteins and contribute to an epigenetic code recognized by proteins involved in regulation of gene expression (Marks *et al*., 2003, 2004; Hooven, *et al*., 2005). In neoplastic cells, where overexpression of different histone deacetylases was frequently detected (for summary see, Bolden *et al*., 2006), the abundance of deacetylated histones is usually associated with DNA hypermethylation and gene silencing (Santini *et al*., 2007). Treatment with histone deacetylase inhibitors induced the reactivation of growth regulatory genes and consequently apoptosis in these cells. One of the histone deacetylase inhibitors, valproic acid (Figure 4), inhibits growth and induces differentiation of human neuroblastoma cells *in vitro* at concentrations ranging from 0.5 to 2 mM that have been achieved in human with no significant adverse effects (Cinatl *et al*., 1996; Hřebačková *et al*., 2009). Ultrastructural features of valproic acid-treated cells were consistent with the neuronal type of differentiation and are associated with decreased expression of N-myc oncoprotein and increased expression of neutral cell adhesion molecule in their membrane. In these cells valproic acid treatment synergized interferon-alpha leading to a massive accumulation of cells in G0/G1-phase. This drug also influences the resistance of neuroblastoma cells to several chemotherapeutics (Blaheta *et al*., 2007). Valproic acid reverts the enhanced adhesion properties of drug-resistant UKF-NB-2, UKF-NB-6 and SKNSH neuroblastoma cells accompanied by diminished N-myc and enhanced p73 protein levels (Blaheta *et al*., 2007). Therefore, this drug may provide an alternative approach to the treatment of drug-resistant neuroblastomas by blocking invasive processes (Blaheta *et al*., 2007).

Recently, combination of histone deacetylase inhibitors valproic acid and/or trichostatin A (Figure 4) with DNA methyltransferase inhibitor 5-azacytidine (Zhu and Otterson, 2003; Chai *et al*., 2008), or with anticancer drugs that act by targeting DNA, vepesid (VP-16), ellipticine, doxorubicin, epirubicin, and cis-platin, enhances their efficacy in several tumour cells (Kim *et al*., 2003; Marchion *et al*., 2005a, b; Catalano *et al*., 2006). Its effect on cytotoxic potential of drugs used for treatment of neuroblastomas has, however, not been evaluated. Our preliminary results suggest that the anticancer activity of ellipticine to neuroblastomas might be synergically increased by these histone deacetylase inhibitors. These preliminary data show that a higher sensitivity of neuroblastoma cells to ellipticine correlated with an increase in formation of covalent ellipticine-derived DNA adducts (unpublished data) that was found to be one of the most important DNA-damaging mechanisms of ellipticine action in neuroblastomas (Poljaková *et al*., 2009). However, the exact mechanisms of these features need further studies. Moreover, the effect of combination of valproic acid and trichostatin A with other DNA-damaging drugs used for neuroblastoma treatments (anthracyclines such as doxorubicin, platinum complexes, cyclophosphamide and etoposide) on growth of neuroblastoma cells and the mechanisms of such a drug combination have not yet been investigated. Hence, these subjects also await further examinations.

## Conclusions

Most of the above mentioned phenomena, which have not been properly explained as yet, are addressed in the grant project of the Grant Agency of Czech Republic (P301/10/0356) solved in our laboratory. Namely, the questions which of DNA-damaging mechanisms of action (non-covalent DNA intercalation, covalent DNA adducts formation, DNA-DNA cross-links and DNA strand-breaks induced by inhibition of topoisomerase II and radical effects) both for drugs currently used for treatment of human neuroblastoma cancers (doxorubicin, platinum complexes, cyclophosphamide and etoposide) and another anticancer agent decreasing growth of neuroblastoma cells *in vitro*, ellipticine, are predominant mechanism(s) responsible for their antitumor action. Their effects in combination with histone deacetylase inhibitors such as valproic acid and/or trichostatin A and mechanisms of such effects are other questions that are also investigated. Such a study will increase our knowledge to explain the proper function of these drugs on the molecular level, which should be utilized for the development of new therapies for neuroblastomas.

Because metabolism of these agents, leading either to covalent modification of DNA by them or to their detoxication, are dependent on expression and activities of enzymes biotransforming drugs such as cytochromes P450 and/or peroxidases for ellipticine (Stiborová *et al*., 2001; 2003a; 2003b; 2004; 2006; 2007a; 2007b; 2008; 2010), etoposide (van Schaik, 2008) or cyclophosphamide (Oesch-Bartlmowicz and Oesch, 2004; van Schaik, 2005; 2008; Wang and Tompkins, 2008) and reductases such as NADPH:cytochrome P450 reductase, carbonyl reductase (secondary-alcohol:NADPH oxidoreductase) and/or NADPH:quinone oxidoreductase for doxorubicin (Merk and Jugert, 1991; Gavelová *et al*., 2008; Lal *et al*., 2010), investigation of their expression levels and activities will be another aim of our research.

A successful solution of the above features seems to have unquestionable practical importance, as it could be utilized for enhancing efficiency of the studied drugs, both of those currently used for neuroblastoma treatment and a novel anticancer agent, ellipticine, utilizing their combine effects with inhibitors of histone deactylases. The results might, therefore, be utilized for the development of new therapies for neuroblastomas. In the case of ellipticine, the results will also be promising for ellipticine utilization in cytochrome P450- and/or peroxidase-mediated gene therapy and for preparation of such ellipticine derivatives that will be appropriate for tumor targeting.
